# Relationship between short-term self-reported dietary magnesium intake and whole blood ionized magnesium (iMg^2+^) or serum magnesium (s-Mg) concentrations

**DOI:** 10.1080/07853890.2023.2195702

**Published:** 2023-04-10

**Authors:** Velarie Y. Ansu Baidoo, Krisha Thiagarajah, Carmen D. Tekwe, Taylor C. Wallace, Nana Gletsu-Miller

**Affiliations:** a Northwestern University Feinberg School of Medicine, Chicago, IL, USA; b School of Public Health-Bloomington, Indiana University, Bloomington, IN, USA; c Department of Nutrition and Food Studies, George Mason University, Fairfax, VA, USA; d Think Healthy Group, Inc, Washington, DC, USA; e Center for Magnesium Education & Research, Pahoa, HI, USA

**Keywords:** Magnesium, diet, nutritional status, dietary assessment, biomarkers, serum magnesium, dietary magnesium, ionized magnesium

## Abstract

**Objective:**

Since we and others have shown that supplemental magnesium raises whole blood ionized magnesium (iMg^2+^) we investigated the relationships between self-reported dietary magnesium intake and concentrations of whole blood iMg^2+^ and serum magnesium (s-Mg).

**Methods:**

We obtained whole blood iMg^2+^ concentrations, as well as s-Mg concentrations, from a pilot, three-arm, randomized, controlled, crossover bioavailability study of magnesium supplements (*n* = 23; 105 measures). Dietary magnesium intake was assessed using three-day food records and the Nutrition Data System for Research (NDSR, University of Minnesota, MN, USA). Whole blood iMg^2+^ was measured with an electrode analyser (NOVA Biochemical, Waltham, MA, USA), whereas s-Mg was measured using atomic absorption spectroscopy. A linear mixed-effects model was employed with dietary magnesium as the outcome variable and iMg^2+^, s-Mg, study treatment and study visit as fixed effects. We adjusted age, gender, race and body mass index covariates.

**Results:**

Values for dietary magnesium, iMg^2+^ and s-Mg were 303.8 ± 118.9 mg/day, 1.3 ± 0.1 mg/dL and 2.2 ± 4.1 mg/dL, respectively. No association was found between dietary magnesium intake and iMg^2+^ −125 ± 176.95 (*p* = .49) or s-Mg −9.33 ± 5.04 (*p* = .08).

**Conclusions:**

Whole blood iMg^2+^ and s-Mg concentrations do not reflect short-term self-reported dietary intake in adults. Further research is needed to determine whether blood biomarkers of magnesium may reflect dietary magnesium intake.

**Key messages**
- Dietary intake of magnesium, a shortfall nutrient, may be objectively measured using blood biomarkers of magnesium.
- Serum magnesium and whole blood iMg^2+^ were not associated with short-term dietary intake of magnesium.

## Introduction

Magnesium is the second most abundant intracellular cation in the human body, and it is an essential mineral involved as a cofactor in over 600 enzymatic reactions [1–3]. Total serum magnesium (s-Mg) is present in three forms: free and ionized (iMg^2+^, 55–70%), protein-bound (20–30%) and anion-bound (5–15%) (e.g. phosphate, citrate, bicarbonate). Of these forms, only iMg^2+^ is active as it is the form that can directly participate in enzymatic reactions. Therefore, while s-Mg concentration is a widely used method to assess magnesium status, it has been suggested that s-Mg may not accurately reflect an individual’s status, due to the hindered availability of bound magnesium to participate in biological processes and the body’s homeostatic control of the nutrient [4]. Moreover, magnesium predominantly resides in the intracellular fluid (∼99%; bones, muscles and soft tissues) with only about 1% in the extracellular space [4]. Limitations of s-Mg as an accurate measure have led researchers to investigate if whole blood iMg^2+^, being the active form, may be used to assess magnesium status.

Nutritional epidemiologic studies have traditionally utilized self-reported dietary intake in research to study the relationship between diet and health outcomes. Assessment methods, including food frequency questionnaires (FFQs), food records and 24-h recall are commonly used for assessing dietary intake [5–7]. Due to the limitations of self-reported dietary intake, nutrition scientists have called for more objective measures [5–7]. Findings from studies by us [8] and others [9,10] have shown an increase in blood iMg^2+^ following acute and chronic intake of supplemental magnesium salts. These findings have led us to speculate that iMg^2+^ may serve as a biomarker of dietary intake of Mg [4,8].

The objective of the current study was to assess associations among blood iMg^2+^, s-Mg and short-term self-reported dietary intake of magnesium. We used data from our pilot clinical trial [8] on the bioavailability of a magnesium chloride supplement, which included dietary and biochemical assessments of status at baseline. We hypothesized that compared to concentrations of s-Mg, concentrations of iMg^2+^ would better correlate with short-term self-reported dietary magnesium intakes.

## Materials and methods

### Study design and population

The design of this study is a retrospective, secondary data analysis of data obtained from a pilot, randomized, placebo-controlled, crossover trial (NCT04139928) that aimed to assess the bioavailability of a single dose of a novel, picometer-sized, magnesium chloride formulation (ReMag®) [8]. For the main study, during each of the three clinic visits, participants consumed a single dose (300 mg) of magnesium chloride or a lemon juice placebo, and pharmacokinetics of absorption was assessed in blood samples over a 24-h period. There was a one-week wash-out between visits. During the week prior to each of the two clinic visits, three-day dietary records were collected (two weekdays and one weekend day). At the start of each study visit, prior to magnesium dosing, we obtained a fasting blood sample from participants. The dietary records and baseline blood sample measures of magnesium were used for the current study. Eligibility included any gender, race, or ethnicity, healthy, body mass index (BMI, 18.5 to 39.9 kg/m^2^) and age 18 to 65 years. To assess healthiness, a study physician reviewed participants’ clinical biochemistries from a comprehensive metabolic panel test to ensure that values were in the normal range. Participants reported not having conditions that affect the cardiac, circulatory and digestive systems, as well as not having renal and hepatic conditions, cancer, diabetes and thyroid diseases. Participants also answered a medical history questionnaire which included medications that they were taking. Study participants refrained from taking Mg supplements within two weeks of study screening and throughout the study. The study was approved by the Purdue University Institutional Review Board (#1802020279), and study participants consented before participation.

### Dietary assessment

Participants reported their dietary intake using a three-day food record (i.e. non-consecutive two-weekdays and one weekend day) before each clinic visit. A food log with instructions on how to complete the dietary record with relevant portion size estimations was given to all participants to assist with accurate reporting. Participants were given a handout to provide details on portion size estimation, a listing of brand names, a detailed food description list and a food preparation list. Study participants brought the three-day food record on each of their clinic study visits and investigators reviewed the records with the participants to ensure accuracy and completion. Subjects were asked to bring any nutritional supplement products they were currently taking. Training personnel entered data from the three-day food records into the Nutrient Database System Research (NDSR; University of Minnesota, Minneapolis, USA) software. At the Purdue Dietary Assessment Center, the NDSR software was used to estimate the daily total energy (kcal) and daily nutrient intake values of foods and recipes based on estimates from the United States Department of Agriculture database [11].

### Biochemical and other assessment methods

Whole blood iMg^2+^ was measured using Nova 8 Electrolyte Analyzer (Nova Biomedical, Waltham, MA, USA) [12]. Serum Mg was measured using an atomic absorption mass spectrometry [13]. Ionized Mg and s-Mg were measured in duplicate at baseline (i.e. before a supplement or placebo was given at each study visit). The BMI of participants was calculated as body weight (kg) divided by height (m^2^) and measured with a scale and stadiometer, respectively.

## Statistical analysis

Categorical (i.e. gender and race) variables were presented as percentages and continuous variables as mean and standard deviation. Exploratory data analysis (i.e. box plots and histograms) was used to summarize the distributions of the study variables. The data were examined for possible outliers and the normality assumption was tested. Diagnostic plots were employed to assess the normality of the residuals, homoscedasticity and case influential statistics. Results were approximately normal.

The linear mixed-effects model was used to examine the influence of varying levels of dietary magnesium intake on whole blood iMg^2+^ and s-Mg. Fixed effects were considered for study visits, study treatment, iMg^2+^ and s-Mg. Random effect was considered for study participants. Age, gender, race and BMI were adjusted as covariates in the model. The restricted maximum likelihood method and the Akaike Information Criterion (AIC) model selection were to select the model with the best-fit [14]. A full model was selected to adjust for race, gender, BMI, study visit and treatment because the chi-square test showed no statistical significance between the full and reduced models (which only adjusted for gender and race). We also corrected the effects of measurement error using a regression calibration approach [15,16]. Using the regression calibration approach, the best prediction of the true usual intake for the self-reported dietary magnesium values was obtained and used as a proxy in analysing the usual intake. All statistical analyses were performed using SAS software (version 9.4; SAS Institute Inc., Cary, NC, USA). Statistical significance was defined as a two-sided *p* value <.05 for all analyses.

## Results

The study characteristics of the participants at visit one is presented in Table 1. Study participants were young adults (28.3 ± 8.7 years) and the mean BMI of the cohort was slightly overweight (25.1 ± 3.0 kg/m^2^) with a range of 21.54 to 33.89 kg/m^2^. The cohort was diverse by gender and race. Mean values for dietary Mg, iMg^2+^ and s-Mg were 303.8 ± 118.9 mg/d, 1.3 ± 0.1 mg/dL and 2.2 ± 4.1 mg/dL, respectively (Table 1). In 19–30 age group, the Recommended Dietary Allowance (RDA) for magnesium intake is 400 and 310 mg/day for males and females, respectively. The standard reference range s-Mg is 1.82–2.30 mg/dL [17,18]. Additionally, a recent consensus for low cut-off points for hypomagnesemia has been published as 2.07 mg/dL [19].

**Table 1.** Characteristics of study participants and the values of magnesium assessment at the first study visit.

Study characteristics	n	Values
Age (years), mean ± SD	23	28.3 ± 8.7
BMI (kg/m^2^), mean ± SD	23	25.1 ± 3.0
Race, %		
White	13	56.5
Hispanic	4	17.4
Asian	3	13.0
Black	3	13.0
Gender, %		
Male	12	52.2
Female	11	47.8
Self-reported dietary intake, mg/d	23	303.8 ± 118.9
Whole blood iMg^2+^, mg/dL	19	1.3 ± 0.1
s-Mg (mg/dL)	22	2.2 ± 4.1

*Notes:* Self-reported demographic data from the study; BMI = Body mass index; SD = standard deviation. there were 105 measures of self-reported dietary intake (primary exposure) made up of 23 participants who had one visit, 20 who had two visits and 14 who had three visits. Values are provided as average means ± standard deviations and percentages for continuous and categorical variables respectfully. Dietary intake was obtained from three-day food records collected prior to each of the three clinical visits. Ionized Mg and serum Mg were obtained from duplicate samples collected at baseline prior to each of the three clinical visits and measured with electroanalyser and atomic absorption spectroscopy, respectively. Divide by 2.43 to convert from mg/dL to mmol/L.

The fixed-effect regression model was used to assess associations among blood iMg^2+^, s-Mg and dietary intake of Mg. The beta-estimate value obtained from our linear mixed models for whole blood iMg^2+^ and s-Mg were −125 ± 176.95, (*F* = 0.50, *p* = .49) and −9.33 ± 5.04, (*F* = 3.43, *p* = .08), respectively (Table 2). We also did not find statistically significant associations between whole blood iMg^2+^ and s-Mg (*p* = .08).

**Table 2.** Association of whole blood iMg^2+^ and s-Mg in the model.

	Estimate ± SE	P>|t|
Whole blood iMg^2+^, mg/dL	−125.40 ± 176.95	0.49
s-Mg, mg/dL	−9.33 ± 5.04	0.08

*Notes:* Results from the linear mixed-effects model adjusted for gender, age, body mass index (BMI), race, study visits and treatment. SE: standard error.

## Discussion

We investigated the relationship between short-term self-reported dietary magnesium intake and both whole blood iMg^2+^ and s-Mg measures. The main findings from this study suggest that there are no relationships between short-term self-reported dietary magnesium intake and both iMg^2+^ and s-Mg concentrations. To our knowledge, no study has reported on the association between dietary magnesium intake from food and whole blood iMg^2+^ levels, and very few studies [20,21] have examined magnesium dietary intake and association with s-Mg. Wei et al. did not find any association between dietary magnesium intake and s-Mg in a population with diabetes [20]. However, in a different study, significant correlations, albeit low, were found between dietary magnesium intake and s-Mg in the study population (*r* = 0.049, *p* = .009) as well as in a subgroup without diabetes (*r* = 0.060, *p* = .006) [20]. Additionally, dietary magnesium intake and s-Mg showed a positive correlation in a Japanese cohort (*r* = 0.28, *p* < .05), however, this study was limited by the use of a single-day food record [21]. Despite findings from us [8] and others [9,10], suggesting that iMg^2+^ responds to supplemental magnesium intake, results from this current analysis did not support its use as a dietary biomarker. One reason for the lack of a correlation between dietary Mg intake and both iMg^2+^ and s-Mg is that when intake of Mg is low, the body releases Mg reserves from the bone to help maintain Mg homeostasis thus limiting the use of iMg^2+^ and s-Mg as a dietary biomarker [22]. Another possible explanation could be that iMg^2+^ may only be a useful marker for supplemental or acute dietary intake of magnesium.

A strength of our study is the use of repeated measures for the assessment of dietary magnesium intake (up to three separate records for each of the three clinic visits per subject), whole blood iMg^2+ ^ and s-Mg (duplicate measures for iMg^2+ ^and s-Mg). Limitations of our study include low sample size, the inherent challenges of self-reported dietary intake that are prone to reliance on memory, estimation of portion size, underreporting of intake and the propagation of error from the dietary database. Despite the measures taken by trained investigators to ensure that participants recorded and estimated intake accurately, we cannot rule out some of these errors. We used a regression calibration method in predicting dietary magnesium intakes to reduce some of the bias of self-reported measures. Finally, the sample size may not be adequately powered to detect small changes in s-Mg and iMg^2+^ levels in response to dietary intake.

## Conclusion

Our findings suggest that whole blood iMg^2+^ and s-Mg levels may not directly reflect short-term dietary magnesium intake in this sample of healthy adults. While whole blood iMg^2+^ may be a good indicator of supplemental magnesium intake, further research is needed to identify objective biomarkers that reflect dietary intake from food. This will enable a better understanding of the relationship between dietary magnesium intake and cardiometabolic disease states.

## Data Availability

Raw data were generated at Indiana University-Bloomington. Derived data supporting the findings of this study are available from the corresponding author (NGM) on request.
